# Progress and trends in the development of therapies for Hutchinson–Gilford progeria syndrome

**DOI:** 10.1111/acel.13175

**Published:** 2020-06-28

**Authors:** Wing‐Fu Lai, Wing‐Tak Wong

**Affiliations:** ^1^ School of Life and Health Sciences The Chinese University of Hong Kong (Shenzhen) Shenzhen China; ^2^ Department of Applied Biology and Chemical Technology Hong Kong Polytechnic University Hong Kong Special Administrative Region China

**Keywords:** Hutchinson–Gilford progeria syndrome, laminopathy, premature aging, treatment

## Abstract

Hutchinson–Gilford progeria syndrome (HGPS) is an autosomal‐dominant genetic disease that leads to accelerated aging and often premature death caused by cardiovascular complications. Till now clinical management of HGPS has largely relied on the treatment of manifestations and on the prevention of secondary complications, cure for the disease has not yet been established. Addressing this need cannot only benefit progeria patients but may also provide insights into intervention design for combating physiological aging. By using the systematic review approach, this article revisits the overall progress in the development of strategies for HGPS treatment over the last ten years, from 2010 to 2019. In total, 1,906 articles have been retrieved, of which 56 studies have been included for further analysis. Based on the articles analyzed, the trends in the use of different HGPS models, along with the prevalence, efficiency, and limitations of different reported treatment strategies, have been examined. Emerging strategies for preclinical studies, and possible targets for intervention development, have also been presented as avenues for future research.

## Graphical abstract

Hutchinson–Gilford progeria syndrome is an autosomal‐dominant genetic disease which has no cure right now. Development of therapies has clinical importance and may provide a window into the mechanism and treatment of physiological aging.

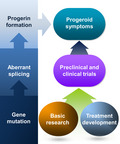

## 1 INTRODUCTION

Hutchinson–Gilford progeria syndrome (HGPS) is a sporadic, autosomal‐dominant genetic disorder (De Sandre‐Giovannoli et al., 2003; Eriksson et al., 2003; Hennekam, 2006; Merideth et al., 2008). Symptoms of this disease include delayed eruption and delayed loss of primary teeth, abnormal skin pigmentation, alopecia, osteoporosis, severe atherosclerosis, nocturnal lagophthalmos, and conductive hearing loss. On average, patients suffering from HGPS often die at around 14.6 years (Hennekam, 2006; Merideth et al., 2008). HGPS is caused by the expression of a mutant lamin A protein, namely progerin. In cells, the *LMNA* gene encodes lamin A, lamin C, lamin CΔ10, and lamin C2 via alternative splicing (Gonzalo, Kreienkamp, & Askjaer, 2017). Lamin A is first synthesized as a prelamin A precursor, which possesses a CAAX motif in its C terminus (Gonzalo et al., 2017). After that, farnesylation occurs, followed by cleavage of the last three residues, with the terminal cysteine being carboxymethylated at last (Prokocimer, Barkan, & Gruenbaum, 2013). The 15 C‐terminal residues of prelamin A are finally cleaved by Zmpste24, leading to the formation of mature lamin A (Gonzalo et al., 2017). In HGPS patients, the process of endoproteolytic cleavage, however, fails to occur, leading to the formation of an aberrant lamin A product which remains to be farnesylated and carboxymethylated (Ahmed, Ikram, Bibi, & Mir, 2018). This leads to diverse abnormalities in nuclear processes and eventually causes organismal malfunction.

Over the years, extensive efforts have been devoted to examining alterations in proteomic and genomic profiles, as well as to deciphering the molecular network that associates the generation of progerin with the occurrence of pathological phenotypes (Benson, Lee, & Aaronson, 2010; Chojnowski et al., 2015, 2020; Kudlow, Stanfel, Burtner, Johnston, & Kennedy, 2008). Comparatively few efforts, however, have targeted specifically at developing treatment strategies for HGPS. In fact, at the moment no treatment is known to be effective to cure HGPS. Clinically, management of the disease relies largely on the treatment of manifestations and on the prevention of secondary complications. For instance, anticongestive therapy is adopted to tackle congestive heart failure (Gordon, Brown, & Collins, 1993). Physical therapy, body bracing, or even reconstructive hip surgery are also used to manage the problem of hip dislocation (Gordon et al., 1993). Although these interventions can ameliorate the symptoms of the disease or improve patients' quality of life, they are mainly palliative in nature. Development of treatment strategies that can authentically treat the disease is, therefore, in dire need. This article reviews systematically the latest development and performance of the treatment strategies reported over the last 10 years.

## 2 LITERATURE SEARCH

### 2.1 Sources

Information used in this article came mainly from an online database search using PubMed and Web of Science. Potentially relevant publications were identified by using the following keywords with different combinations using Boolean operators “AND” and “OR” in appropriate ways: “progeria,” “progeroid,” “premature aging,” and “premature ageing.” References from these articles were manually searched and cross‐referenced to identify additional relevant publications. In order to understand the most recent trend and research progress, only articles published between January 2010 and December 2019 were retrieved. A total of 1,906 entries were identified.

### 2.2 Study selection

Based on the information available in the titles and abstracts of the publications, each of the entries was screened individually. Articles that were deemed relevant were further examined based on the full text to identify eligible studies for inclusion in this review. Studies considered eligible were those that could meet the following inclusion criteria:

- The article involves an intervention administered to an in vitro (and/or in vivo) model of HGPS.
- The intervention reported by the article aims at ameliorating, manipulating, or eradicating at least one pathological symptom of HGPS, or preventing that symptom from occurrence.
- The article collects and reports primary data regarding the performance of the intervention.
- The article discloses details of the experimental procedures.
- The article is written in English.

**FIGURE 1:**
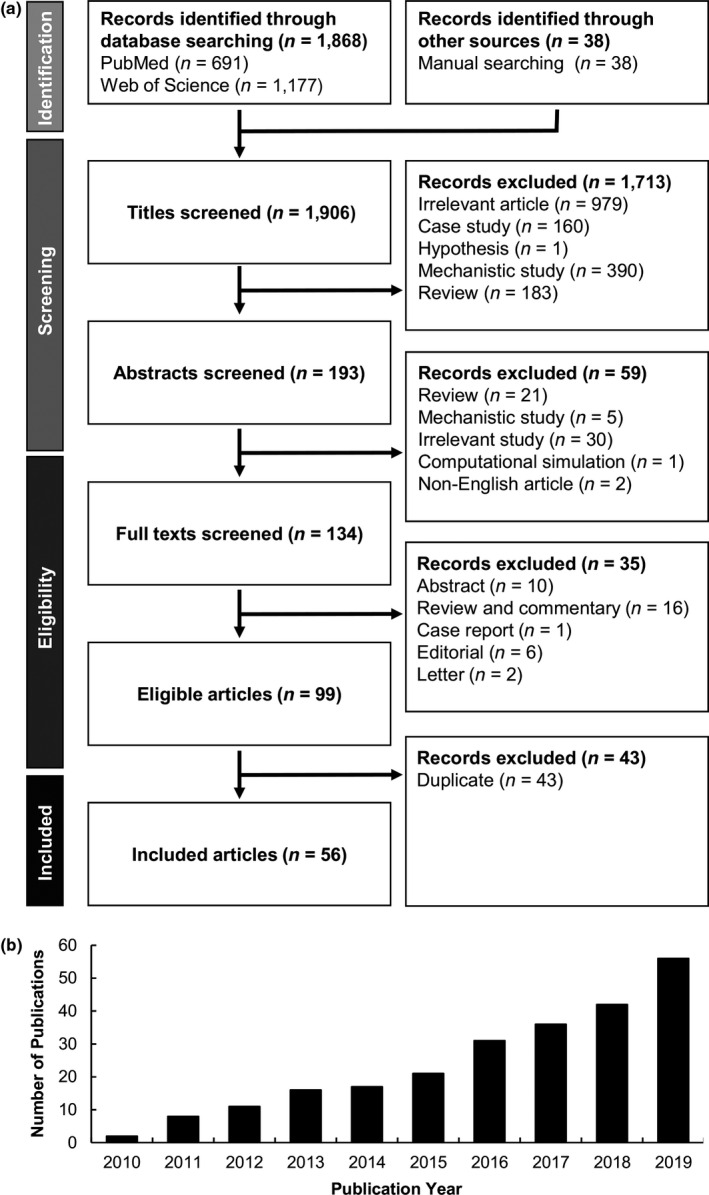
(a) Flow diagram depicting the review process. (b) The cumulative number of included articles retrieved from each year in the period under review

An article was excluded if it did not meet at least one of the five criteria above, if the paper was retracted at a later point within the analyzed period, or if it was a commentary, letter, editorial, abstract, dissertation, corrigendum, erratum, or case study. Of the 1,906 articles, 99 studies met all inclusion criteria. Excluding the duplicates, a total of 56 studies were included for further analysis. The whole process of article selection and evaluation is depicted in Figure 1. Based on the selected articles, the trends in the use of disease models, the types of treatment strategies developed, the methods of intervention execution, the types of molecular targets exploited for treatment development, the performance of reported treatment strategies in preclinical and clinical trials, as well as emerging strategies for future preclinical studies were explored and revisited.

## 3 USE OF PROGERIA MODELS FOR TREATMENT EVALUATION

As far as the use of models for treatment evaluation is concerned, in vitro models are the most intensively adopted ones. Many of the analyzed studies (48%) have evaluated the efficacy of the treatment only at the in vitro level, though some studies (25%) have reported the performance of the treatment only in the preclinical or clinical context whereas others (27%) have simultaneously reported the efficacy of the treatment both in vitro and in vivo (27%). Among the studies that have evaluated the treatment only in vitro, fibroblasts from patients suffering from HGPS have been extensively used (>85%). Most of the studies have obtained progeria fibroblasts directly from the Coriell Cell Repositories or the Progeria Research Foundation Cell and Tissue Bank, although some studies (≈30%) have used primary fibroblasts attained directly from patients for their evaluation. The comparatively high rate of use of cell lines may be due to the high accessibility and commercial availability of those cells. Apart from HGPS fibroblasts from humans, other cell types have been employed. Examples of these cell types include the stem progenitor cells derived from hindlimb skeletal muscles of *Zmpste24^−/−^* mice (Kawakami et al., 2019), marrow‐isolated adult multilineage inducible (MIAMI) cells (Pacheco et al., 2014), patient‐specific induced pluripotent stem cells (Liu et al., 2011), and cells fabricated by introducing the heterozygous *LMNA^G608G/+^* mutation into human embryonic stem cells (hESCs) followed by directed differentiation into human mesenchymal stem cells (hMSCs) (Geng et al., 2019).

Among the studies involving preclinical or clinical evaluation, around 14% of them have reported or evaluated the performance of the reported treatment strategy in human subjects. All of the remaining studies have used mouse models for treatment evaluation. Over the years, various transgenic mouse models of HGPS have been established. One of them is the *Zmpste24^−/−^* mice, which emerged in the literature in the early 2000s (Bergo et al., 2002; Pendas et al., 2002). Although these mice show progeroid features (including muscular dystrophy, lipodystrophy, and premature death) (Mayoral, Bárcena, & López‐Otín, 2018), the mutation in their genome is different from that typically observed in the genome of HGPS patients. To address this problem, mouse models carrying the c.1827C>T; p.G609G mutation, which is equivalent to the c.1824C>T; p.G608G mutation in humans, were established (Osorio et al., 2011). These mice phenocopy most of the clinical symptoms of HGPS and are extensively used for the study of the disease. Although *Lmna^G609G/G609G^* and *Zmpste24^−/−^* mice have been adopted by almost 80% of the analyzed studies involving the use of mouse models, other types of transgenic mice have also been used for treatment evaluation. For instance, one study has used transgenic mice expressing progerin with a FLAG epitope tag in epidermal keratinocytes (Wang, Ostlund, & Worman, 2010), whereas another study has used a tetracycline‐inducible transgenic mouse line in which progerin is expressed in the keratin 5 (K5)‐positive compartment of the skin (Aguado et al., 2019). Other models that have been adopted include C57BL/6J mice with vascular smooth muscle cell (VSMC)‐specific progerin expression (Hamczyk et al., 2019), immunodeficient mice to whom MSCs are implanted (Kubben et al., 2016), mice carrying copies of the OSKM polycistronic cassette and the rtTA transactivator (Ocampo et al., 2016), and mice with osteoblast‐ and osteocyte‐specific inducible transgenic expression of the most common HGPS mutation (Strandgren et al., 2015).

Here, it is worth highlighting that, although the use of mouse models has streamlined the evaluation of the treatment performance, mice are still distant from mammals evolutionarily. Genetic and physiological differences between mouse models and human patients may at the end undermine the transferability of data from preclinical studies to clinical trials. This problem has been noted by one of the studies, which has found that *Lmna^G609G/G609G^* mice show phenotypes (e.g., a decline in the heart rate, and impairment in the gastrointestinal function) that are not typical in human patients (Beyret et al., 2019). This raises concerns on the faithfulness of existing progeria models in simulating the situation in a human body. Development and optimization of in vivo models for more accurately predicting the clinical performance of experimental therapeutic agents should continue to be prioritized for research on HGPS. This is especially true when optimal doses, dose frequencies, administration routes, and adverse drug effects are evaluated for a treatment modality to be transposed to patients. Nevertheless, compared to mere in vitro studies, in vivo evaluation enables the bioavailability and pharmacokinetics of the administered agent to be more comprehensively investigated and hence can provide more useful information for reference of subsequent clinical examination and use.

## 4 PHARMACOLOGICAL TREATMENT FOR HUTCHINSON–GILFORD PROGERIA SYNDROME

Over the last ten years, extensive efforts (94.6%) have been directed to developing genetic/pharmacological interventions, although few studies (5.4%) have devoted to treating HGPS by using protein therapy, diet control, or fecal microbiota therapy (Table 1). Among different modalities of pharmacological treatment, protein farnesyltransferase inhibitors (FTIs) are the most commonly used therapeutic agents, being adopted in 22.5% of studies involving pharmacological treatment. The rationale behind is supported by the fact that the accumulation of farnesyl‐prelamin A is one of the major mechanisms disrupting the scaffolding function of the nuclear lamina (Fong et al., 2006), resulting in misshapen nuclei. By inhibiting protein farnesylation, disruption in nuclear scaffolding, as well as the symptoms of progeria, is expected to be ameliorated (Fong et al., 2006). This has been supported by the observation that treatment with lonafarnib improves the bone structure, the audiological status, and the neurologic function of children with HGPS (Gordon et al., 2012; Ullrich et al., 2013) and reduces the mortality rate (Gordon et al., 2018). This demonstrates the clinical potential of using lonafarnib monotherapy in the treatment of HGPS.

**TABLE 1:** Different regimens reported for the treatment of HGPS

Type	Treatment regimen	Ref.
Pharmacological treatment	Treatment with FTI‐276	Wang et al. (2010)
	Treatment with FTI‐277	Pacheco et al. (2014)
	Treatment with a combination of pravastatin and zoledronate	Wang et al. (2010)
	Treatment with a combination of FTI‐277 and GGTI‐2147	Mehta et al. (2011)
	Treatment with rapamycin	Cao et al. (2011); Cenni et al. (2011); Kawakami et al. (2019)
	Treatment with leptomycin B	Garcia‐Aguirre et al. (2019)
	Treatment with various regimens containing FTI‐277 or rapamycin	Bikkul et al. (2018)
	Treatment with baricitinib	Liu et al. (2019)
	Treatment with a combination of levamisole and ARL67156	Villa‐Bellosta (2019)
	Treatment with resveratrol	Liu et al. (2012); Strandgren et al. (2015)
	Treatment with Y‐27632	Kang et al. (2017)
	Treatment with a combination of lonafarnib and sulforaphane	Gabriel et al. (2017)
	Treatment with N6‐isopentenyladenosine	Bifulco et al. (2013)
	Treatment with a combination of all‐trans retinoic acid and rapamycin	Pellegrini et al. (2015)
	Treatment with sulforaphane	Gabriel et al. (2015)
	Treatment with methylene blue	Xiong et al. (2016)
	Treatment with JH4	Lee, Jung, et al. (2016)
	Treatment with 1α,25‐dihydroxyvitamin D3	Kreienkamp et al. (2016)
	Treatment with lonafarnib/pravastatin/zoledronic acid triple therapy	Gordon et al. (2016)
	Treatment with temsirolimus	Gabriel et al. (2016)
	Treatment with metformin	Egesipe et al. (2016); Park and Shin (2017)
	Treatment with a combination of rapamycin and dimethylsulfoxide	Akinci et al. (2017)
	Treatment with lonafarnib monotherapy	Gordon et al. (2012); Gordon et al. (2018); Ullrich et al. (2013)
	Treatment with MG132	Harhouri et al. (2017)
	Treatment with small‐molecule NRF2‐activating agents	Kubben et al. (2016)
	Treatment with N‐acetyl cysteine (NAC)	Kubben et al. (2016); Richards et al. (2011)
	Treatment with ABT‐737	Ovadya et al. (2018)
	Treatment with quercetin	Geng et al. (2019)
	Treatment with vitamin C	Geng et al. (2019)
	Treatment with S‐adenosyl methionine (SAMe)	Mateos et al. (2018)
	Treatment with BRL37344	Ho et al. (2019)
	Treatment with spermidine	Ao et al. (2019)
	Treatment with CP‐466722	Kuk et al. (2019)
	Treatment with tauroursodeoxycholic acid	Hamczyk et al. (2019)
	Treatment with sodium pyrophosphate tetrabasic decahydrate	Villa‐Bellosta et al. (2013)
	Treatment with KU55933 ATM inhibitor	Osorio et al. (2012)
	Treatment with sodium salicylate	Osorio et al. (2012)
Protein therapy	Treatment with recombinant IGF‐1	Marino et al. (2010)
Microbiota therapy	Fecal microbiota transplantation	Barcena et al. (2019)
Nucleic acid therapy	Genetic manipulation to deplete methyltransferase Suv39h1	Liu et al. (2013)
	Genetic manipulation to reduce the isoprenylcysteine carboxyl methyltransferase (ICMT) expression and activity	Ibrahim et al. (2013)
	Genetic manipulation to overexpress SIRT6	Endisha et al. (2015)
	Genetic manipulation to knockdown the phospholipase A2 receptor	Griveau et al. (2018)
	Genetic manipulation to inhibit DNA damage response at telomeres	Aguado et al. (2019)
	Genetic manipulation to disrupt the last part of the *LMNA* gene and to impede lamin A/progerin production without affecting the production of lamin C	Santiago‐Fernandez et al. (2019)
	Genetic manipulation to enhance the activity of telomerase	Li et al. (2019)
	Genetic manipulation to cause lamin A/progerin‐specific transcriptional interference or RNA destabilization	Beyret et al. (2019)
	Genetic manipulation to enhance caNRF2 expression	Kubben et al. (2016)
	Genetic manipulation to knockdown CAND1 expression	Kubben et al. (2016)
	Genetic manipulation to inhibit pathogenic *LMNA* splicing	Harhouri et al. (2016); Osorio et al. (2011)
	Genetic manipulation to enhance lamin C production at the expense of prelamin A	Lee, Nobumori, et al. (2016)
	Genetic manipulation to inhibit NF‐kB activation	Osorio et al. (2012)
	Genetic manipulation to correct or silence the HGPS mutation	Liu et al. (2011); Strandgren et al. (2015)
	Genetic manipulation to express Yamanaka factors	Ocampo et al. (2016)
Diet control	Methionine restriction	Barcena et al. (2018)

Despite this promising potential, owing to the cardiotoxicity (which is caused by the occurrence of nonfarnesylated prelamin A accumulation) caused by FTIs, long‐term administration of FTI‐based therapies has raised safety concerns (Davies et al., 2010). In addition, due to alternative prenylation possibly undergone by prelamin A and by progerin/LAΔ50 under the action of geranylgeranyltransferases during farnesyltransferase inhibition (Varela et al., 2008), concerns have been raised on the efficiency of monotherapy mediated by FTIs alone. Previously, a study has reported that treatment with a combination of statins and aminobisphosphonates effectively inhibits both farnesylation and geranylgeranylation of progerin and prelamin A, leading to an improvement in progeroid phenotypes of *Zmpste24^−/−^* mice (Varela et al., 2008). This may partially explain the phenomenon that around 30% of the analyzed studies involving the use of FTIs have chosen to co‐administer inhibitors of progerin prenylation. Examples of these inhibitors include pravastatin (Gordon et al., 2016), zoledronic acid (Gordon et al., 2016), and GGTI‐2147 (Mehta, Eskiw, Arican, Kill, & Bridger, 2011). Among them, the combined use of lonafarnib with pravastatin and zoledronic acid has been examined by one of the clinical trials reported over the last ten years (Gordon et al., 2016). Upon administration of the triple therapy, 71.0% of the participants have achieved the primary outcome success, which has been predefined as an improvement in the per‐patient rate of weight gain or in carotid artery echodensity (Gordon et al., 2016). Compared with lonafarnib monotherapy, triple therapy has given additional bone mineral density benefits to patients, though additional cardiovascular benefits led by the triple therapy has been found to be minimal (Gordon et al., 2016). This suggests the potential use of a cocktail regimen to enhance the treatment performance.

Apart from the aforementioned FTI‐based therapies that have been evaluated clinically, there are few other agents adopted in pharmacological treatment in preclinical trials. These include resveratrol (Liu et al., 2012), levamisole (Villa‐Bellosta, 2019), ARL67156 (Villa‐Bellosta, 2019), MG132 (Harhouri et al., 2017), JH4 (Lee, Jung, et al., 2016), NRF2‐activating agents (oltipraz, CPDT, TAT‐14, AI‐1) (Kubben et al., 2016), ABT‐737 (Ovadya et al., 2018), sodium salicylate (Osorio et al., 2012), β3‐AR agonist (Ho et al., 2019), spermidine (Ao et al., 2019), tauroursodeoxycholic acid (TUDCA) (Hamczyk et al., 2019), and sodium pyrophosphate tetrabasic decahydrate (Villa‐Bellosta et al., 2013). They have been reported to show therapeutic effects in mouse models. For example, in *Zmpste24^−/−^* mice, not only has treatment with resveratrol rescued the adult stem cell (ASC) decline and slowed down body weight loss, but it has also improved the trabecular bone structure and mineral density and has prolonged the lifespan of the mice (Liu et al., 2012). In *Lmna*‐mutant mice, treatment with the autophagy‐activating agent, MG132, has been found to reduce the levels of progerin and SRSF‐1 (Harhouri et al., 2017). Administration of JH4 has also led to a significant improvement in progeroid phenotypes and an extension of the lifespan (Lee, Jung, et al., 2016). More recently, combined treatment with ATP, levamisole, and ARL67156 has been shown to prevent vascular calcification and to extend the longevity of the *Lmna*‐mutant mice by 12% (Villa‐Bellosta, 2019). Further optimization of some of these agents might be needed to promote the treatment efficiency in practice. This is partly exemplified by the case of resveratrol, whose efficiency in SIRT1 activation is largely limited by poor bioavailability and by variable dose‐dependent effects. Nevertheless, due to the therapeutic effects displayed by these reported agents, some of them may become candidates for evaluation in clinical trials in the future development of pharmacological treatment.

## 5 USE OF NUCLEIC ACID THERAPY TO TACKLE PROGERIA

Among the analyzed studies reporting preclinical and clinical trials, over 40% of them have exploited the use of nucleic acid therapy to treat HGPS. Strategies reported for the execution of the therapy include prenatal genetic manipulation, antisense oligonucleotide therapy, CRISPR/Cas9‐based therapy, and ex vivo genetic manipulation (Table 2). Implementation of most of these strategies, however, requires the intervention to be administered before birth. For instance, whole‐body knockout of *Pla2r1* in progeroid mice has been achieved by breeding *Pla2r1^−/−^* mice with *Zmpste24^−/−^* mice (Griveau et al., 2018). The same also applies to the manipulation of isoprenylcysteine methylation, which has been attained by breeding *Icmt^hm/hm^* mice with *Zmpste24^−/−^* mice (Ibrahim et al., 2013). Due to the lack of technologies at the moment to genetically modify cells and tissues throughout a postnatal body (Lai, 2011, 2012, 2013; Lai, Lin, & Wong, 2019), the technical viability of translating the corresponding research findings into a practicable intervention for patients suffering from HGPS is low. The same problem also occurs in CRISPR/Cas9‐based therapy reported by Beyret et al. (2019) and in antisense oligonucleotide therapy reported by Aguado et al. (2019). The former study has administered the therapy to progeroid mice which have been genetically modified to be hemizygous for a constitutively active Cas9 transgene, whereas the latter study has injected anti‐teloG or anti‐teloC systemically into mice at embryonic day 17 to suppress the DNA damage response specifically at telomeres, followed by administration of additional antisense oligonucleotide therapy starting at postnatal day 2. Because these interventions involve prior genetic modification of an individual, they can hardly be executed in practice. In addition, owing to the ethical issues caused by genetic editing of embryos, the applicability of these strategies in even preventing the occurrence of HGPS may raise ethical and technical concerns.

**TABLE 2:** Strategies of nucleic acid therapy reported for preclinical HGPS treatment

Strategy	Objective	Effects	Ref.
Prenatal genetic manipulation	To deplete methyltransferase Suv39h1	Loss of Suv39h1 in progeroid mice delayed body weight loss, increased bone mineral density, and extended lifespan	Liu et al. (2013)
	To reduce the expression and activity of isoprenylcysteine carboxyl methyltransferase (ICMT)	A hypomorphic allele of ICMT increased body weight, normalized grip strength, and extended the lifespan of progeroid mice	Ibrahim et al. (2013)
	To knockdown the phospholipase A2 receptor	Whole‐body knockout of *Pla2r1* in progeroid mice ameliorated premature aging phenotypes (including rib fractures and the decline in bone content)	Griveau et al. (2018)
	To inhibit the NF‐κB pathway	The therapy increased body weight and extended the lifespan of the mouse model. In addition, after treatment, the spleen of the mouse model showed normal lymphoid follicles. The thymus of the mouse model also displayed normal tissue mass, cellularity, and architecture	Osorio et al. (2012)
	To overexpress Yamanaka factors	The therapy ameliorated organismal phenotypes associated with HGPS	Ocampo et al. (2016)
	To silence the HGPS mutation	The therapy normalized the bone morphology and mineralization in the mouse model, in which osteoblast‐ and osteocyte‐specific inducible transgenic expression of the HGPS mutation had been incorporated. It also normalized dentinogenesis, and increased the number of osteocytes in remodeled bone.	Strandgren et al. (2015)
Antisense oligonucleotide therapy	To inhibit DNA damage response	Treatment with sequence‐specific telomeric antisense oligonucleotides led to a significant reduction in the number of telomere dysfunction‐induced foci in progeroid mice. Restoration of homeostatic proliferation in the suprabasal layer of the skin of the mice was also observed	Aguado et al. (2019)
	To prevent pathogenic *Lmna* splicing	The therapy reduced the accumulation of progerin, ameliorated progeroid phenotypes, and extended the lifespan of progeroid mice	Osorio et al. (2011)
	To increase lamin C production at the expense of prelamin A	The therapy ameliorated the aortic pathology observed in *Lmna^G609G/G609G^* mice	Lee, Nobumori, et al. (2016)
Ex vivo treatment of cells before implantation	To reactivate the NRF2 pathway by knocking down CAND1	The therapy could not only restore the in vivo viability of MSCs obtained from the differentiation of the induced pluripotent stem cells (iPSCs) derived from HGPS fibroblasts, but could also decrease the reactive oxygen species (ROS) level and could rescue nuclear defects in those cells	Kubben et al. (2016)
CRISPR/Cas9‐based therapy	To impede lamin A/progerin production	The therapy led to a significant reduction in the number of progerin‐positive nuclei in the liver, heart and skeletal muscles of the progeroid mice	Santiago‐Fernandez et al. (2019)
	To cause lamin A/progerin‐specific transcriptional interference or RNA destabilization	The therapy suppressed epidermal thinning and dermal fat loss, ameliorated the degeneration of vascular smooth muscle cells of the aortic arch, attenuated the development of bradycardia, and increased the median survival rate of progeroid mice	Beyret et al. (2019)

Among all the analyzed preclinical trials adopting therapeutic nucleic acids to treat HGPS, strategies that show higher practicability in the clinical context are antisense morpholino‐based therapy that prevents pathogenic *LMNA* splicing (Osorio et al., 2011) and antisense oligonucleotide therapy that increases lamin C production at the expense of prelamin A (Lee, Nobumori, et al., 2016). These two therapies have been executed in vivo via systemic injection of the therapeutic nucleic acids and hence can be possibly translated into treatment of HGPS patients in reality, even though off‐targets still have to be evaluated. The treatment modality reported by Santiago‐Fernandez and coworkers also shows a possibility for direct translation into a therapy (Santiago‐Fernandez et al., 2019). The study has used an adeno‐associated virus serotype 9 (AAV9) as a delivery system, partly owing to the comparatively broad tissue tropism and the high safety of the AAV vector (Santiago‐Fernandez et al., 2019). *Staphylococcus aureus* Cas9 nuclease has been used, and a single‐guide RNA (sgRNA) molecule with a 5′‐NNGRRT protospacer‐adjacent motif (PAM) sequence has been designed to target *LMNA* exon 11 upstream of the HGPS mutation (Santiago‐Fernandez et al., 2019). Upon packaging of the vector, 2 × 10^11^ AAV9 genome copies have been injected intraperitoneally into *Lmna^G609G/G609G^* mice (Santiago‐Fernandez et al., 2019). Because this strategy requires no pregenetic modification of an individual, it can be easily applied to patients suffering from HGPS. However, partly due to the lower tropism in organs such as lung, kidney, and aorta, the treatment has been found to show little effect in reducing the number of progerin‐positive nuclei in these organs (Santiago‐Fernandez et al., 2019). In addition, upon administration of the vector, the global reduction in the level of mRNA of progerin was too low to be properly detected (Santiago‐Fernandez et al., 2019). This suggests that the efficiency of whole‐body genome editing mediated by the vector still has ample space for enhancement.

## 6 EMERGING STRATEGIES FOR PRECLINICAL TRANSITION

Apart from the treatment strategies that have been verified in preclinical and clinical trials as mentioned above, there are strategies that have only been tested in the in vitro context (Table 3). Among all of the analyzed studies that have reported in vitro‐tested treatment strategies, four articles have adopted therapeutic nucleic acids to mediate intervention execution. Two have used nonviral reagents [viz., the Endoporter system (Harhouri et al., 2016) and lipofectamine (Li et al., 2019)] to deliver the nucleic acid, whereas the other two studies have used viral vectors. One of the latter two studies has used a lentiviral vector to mediate *SIRT6* overexpression (Endisha et al., 2015), and the other one has employed a helper‐dependent adenoviral vector (HDAdV) for the correction of different mutations spanning a substantially large region of the *LMNA* gene (Liu et al., 2011). Different from the Endoporter system and lipofectamine whose applications are confined to the laboratory context, viral vectors have a track record of clinical use (Hacein‐Bey‐Abina et al., 2008; Kohn, Sadelain, & Glorioso, 2003; McCormack & Rabbitts, 2004) and hence may enable the therapy to be more readily transitioned into future clinical practice. Furthermore, compared to the adenoviral vector which enables mainly transient transgene expression and may require repeated administration for long‐term effects, the lentiviral vector enables stable expression of the transgene and is, therefore, a candidate for use in genomic correction in HGPS patients. Despite this, several issues have to be settled before the strategy can be applied clinically. One is the safety issue. In fact, stable transgene expression brought about by the use of the lentiviral vector always comes with insertional mutagenesis. This problem has raised concerns since the early 2000s when two patients suffering from severe combined immunodeficiency‐X1 (SCID‐X1) had developed acute lymphoblastic leukemia (T‐ALL) after recipient of gene therapy mediated by the retroviral vector (Hacein‐Bey‐Abina et al., 2008; Kohn et al., 2003; McCormack & Rabbitts, 2004). Such concerns have been further accentuated when other instances of preneoplastic or truly neoplastic cell expansion have been reported to be associated with gene therapy of Wiskott–Aldrich syndrome (WAS) (Boztug et al., 2010) and of X‐linked chronic granulomatous diseases (Ott et al., 2006). There is still a long way to go before a safe vector can be developed to mediate stable transgene expression to tackle HGPS.

**TABLE 3:** Treatment strategies verified only in vitro for tackling HGPS

Type of agents	Strategy	Effects	Ref.
Small‐molecule compound	Treatment with inhibitors to prevent progerin farnesylation and geranylgeranylation	Treatment of progeria cells with the farnesyltransferase inhibitor FTI‐277 and the geranylgeranyltransferase inhibitor GGTI‐2147 successfully corrected the disease‐associated changes in chromosome territory positions and chromosome dynamics	Mehta et al. (2011)
	Treatment with rapamycin alone	Rapamycin treatment of progeria cells lowered the levels of progerin and wild‐type prelamin A. It could also increase the relative expression of ZMPSTE24, which is a prelamin A endoprotease	Cenni et al. (2011)
		Rapamycin treatment of progeria cells abolished nuclear blebbing, delayed the onset of cellular senescence, and enhanced progerin degradation	Cao et al. (2011)
		Treatment of muscle‐derived stem/progenitor cells obtained from progeroid mice with rapamycin improved the capacity of myogenic and chondrogenic differentiation, and reduced the extent of apoptosis and senescence	Kawakami et al. (2019)
	Treatment with a farnesyltransferase inhibitor alone	Treatment of GFP‐progerin marrow‐isolated adult multilineage inducible MIAMI cells with FTI‐277 reduced the number of abnormal nuclei, decreased the stiffness in both cytoplasmic and nuclear regions, and enhanced the self‐renewal capacity of those cells	Pacheco et al. (2014)
	Treatment with rapamycin and all‐trans retinoic acid	Treatment of progeria cells with rapamycin, along with all‐trans retinoic acid, reduced the levels of progerin and prelamin A, and increased the lamin A to progerin ratio.	Pellegrini et al. (2015)
	Treatment with rapamycin and DMSO	Treatment of progeria cells with DMSO and rapamycin ameliorated nuclear shape abnormalities	Akinci et al. (2017)
	Treatment with rapamycin and a farnesyltransferase inhibitor	Treatment of progeria cells with the farnesyltransferase inhibitor (viz., FTI‐277) and rapamycin restored the genome organization in progeria cells and improved the ability of the cells to repair damaged DNA	Bikkul et al. (2018)
	Treatment with N6‐isopentenyladenosine	Treatment of progeria cells with N6‐isopentenyladenosine ameliorated nuclear shape abnormalities and led to a redistribution of prelamin A away from the inner nuclear envelope	Bifulco et al. (2013)
	Treatment with sulforaphane	Treatment of progeria cells with sulforaphane enhanced progerin clearance, and reduced the extent of DNA damage associated with HGPS	Gabriel et al. (2015)
	Treatment with methylene blue	Treatment of progeria cells with methylene blue alleviated mitochondrial defects caused by HGPS, rescued nuclear shape abnormalities and perinuclear heterochromatin loss, and corrected misregulated gene expression	Xiong et al. (2016)
	Treatment with 1α,25‐dihydroxyvitamin D3	Treatment of progeria cells with 1α,25‐dihydroxyvitamin D3 reduced progerin production, and alleviated some of the disease phenotypes, including nuclear morphological abnormalities, DNA repair defects, and premature senescence	Kreienkamp et al. (2016)
	Treatment with temsirolimus	Treatment of progeria cells with temsirolimus decreased the progerin level, enhanced cell proliferation, and reduced the number of misshapen nuclei	Gabriel et al. (2016)
	Treatment with metformin	Treatment of MSCs derived from progeria fibroblasts with metformin led to a reduction in progerin expression, and ameliorated nuclear shape abnormalities	Egesipe et al. (2016)
		Treatment of progeria cells with metformin delayed cell senescence caused by HGPS, reduced ROS production, and decreased the number of DNA damage foci	Park and Shin (2017)
	Treatment with the ROCK inhibitor	Treatment of progeria cells with the ROCK inhibitor Y‐27632 decreased the number of misshapen nuclei and the frequency of DNA double‐strand breaks	Kang et al. (2017)
	Treatment with a farnesyltransferase inhibitor and sulforaphane	Treatment of progeria cells with lonafarnib and sulforaphane enhanced progerin clearance, prevented prelamin A accumulation, ameliorated nuclear shape abnormalities, and reduced the number of DNA damage foci	Gabriel et al. (2017)
	Treatment with baricitinib	Treatment of progeria cells with baricitinib restored cellular homeostasis, delayed cell senescence, and reduced the expression of proinflammatory markers	Liu et al. (2019)
	Treatment with leptomycin B	Treatment of progeria cells with leptomycin B reduced the number of senescent cells, ameliorated nuclear shape abnormalities, and rescued the loss of heterochromatin	Garcia‐Aguirre et al. (2019)
	Treatment with N‐acetyl cysteine (NAC)	Treatment of progeria cells with NAC rescued the ability to repair double‐strand breaks, and decreased the population‐doubling time	Richards et al. (2011)
	Treatment with vitamin C and/or quercetin	Treatment of HGPS hMSCs with vitamin C and/or quercetin inhibited progerin production, decreased the population‐doubling time, decreased senescence‐associated β‐galactosidase positivity, and increased the proliferative ability of the cells	Geng et al. (2019)
	Treatment with S‐adenosyl methionine (SAMe)	Treatment of progeria cells with SAMe increased the proliferative capacity of the cells, and decreased senescence‐associated β‐galactosidase positivity	Mateos et al. (2018)
	Treatment with CP‐466722	Treatment of progeria cells with CP‐466722 induced mitochondrial functional recovery, reduced progerin accumulation, and ameliorated nuclear defects	Kuk et al. (2019)
Therapeutic nucleic acid	Lentiviral infection for overexpression of SIRT6	Overexpression of SIRT6 in progeria cells led to a reduction in the frequency of SA‐β‐gal positivity, and reduced the number of misshapen nuclei	Endisha et al. (2015)
	Transduction with an adenoviral vector for the correction of the *LMNA* mutation	Transduction of iPSCs derived from HGPS fibroblasts with the viral vector restored the expression of wild‐type lamin A. abolished progerin expression, decelerated senescence, and ameliorated nuclear shape abnormalities	Liu et al. (2011)
	Treatment with morpholino antisense oligonucleotides for progerin downregulation	Antisense‐based progerin downregulation reduced the accumulation of progerin and/or other truncated prelamin A isoforms, ameliorated nuclear shape abnormalities, and reduced senescence in HGPS‐like patients' cells	Harhouri et al. (2016)
	Transfection with human telomerase reverse transcriptase (hTERT) mRNA	Transfection of short telomere‐containing progeria cells with hTERT mRNA increased the proliferative capacity and lifespan of the cells, reduced the level of senescence, and ameliorated nuclear shape abnormalities	Li et al. (2019)

Compared to therapeutic nucleic acids, more studies have adopted small‐molecule compounds to mediate the treatment. Some of the compounds whose possible therapeutic effects on HGPS have been verified in vitro, but not yet in vivo, include the ATM inhibitor (Kuk et al., 2019), rapamycin (Cao et al., 2011; Cenni et al., 2011; Kawakami et al., 2019), all‐trans retinoic acid (Pellegrini et al., 2015), dimethyl sulfoxide (Akinci et al., 2017), N6‐isopentenyladenosine (Bifulco et al., 2013), sulforaphane (Gabriel, Roedl, Gordon, & Djabali, 2015; Gabriel, Shafry, Gordon, & Djabali, 2017), methylene blue (Xiong et al., 2016), 1α,25‐dihydroxyvitamin D3 (Kreienkamp et al., 2016), temsirolimus (Gabriel, Gordon, & Djabali, 2016), metformin (Egesipe et al., 2016; Park & Shin, 2017), Y‐27632 (Kang et al., 2017), baricitinib (Liu, Arnold, Henriques, & Djabali, 2019), leptomycin B (Garcia‐Aguirre et al., 2019), N‐acetyl cysteine (NAC) (Richards, Muter, Ritchie, Lattanzi, & Hutchison, 2011), vitamin C (Geng et al., 2019), quercetin (Geng et al., 2019), and S‐adenosyl methionine (SAMe) (Mateos et al., 2018). Due to the fact that, upon administration to a body, drug molecules may encounter different physiological events (ranging from the clearance by the reticuloendothelial system to the interactions with diverse blood components) which are absent in the in vitro context but can diminish the chance of the molecules to reach tissues for action in practice, further studies in preclinical and clinical trials to determine the pharmacokinetic profiles of these in vitro‐tested agents are required before they can be deemed therapeutic to HGPS.

## 7 TARGETS FOR INTERVENTION DEVELOPMENT

To develop a treatment regimen, proper selection of a biological target is needed. Targets adopted by the analyzed studies are listed in Table 4, which reveals that treatment of HGPS can be executed at multiple levels. Most of the agents target the production and posttranslational processing (Beyret et al., 2019; Bifulco et al., 2013; Bikkul et al., 2018; Egesipe et al., 2016; Gordon et al., 2012, 2016, 2018; Harhouri et al., 2016; Kreienkamp et al., 2016; Lee, Nobumori, et al., 2016; Lee, Jung, et al., 2016; Liu et al., 2011; Mehta et al., 2011; Osorio et al., 2011; Pacheco et al., 2014; Pellegrini et al., 2015; Santiago‐Fernandez et al., 2019; Ullrich et al., 2013; Wang et al., 2010), as well as the downstream action [e.g., NF‐κB signaling (Osorio et al., 2012), NRF2 pathway (Gabriel et al., 2015, 2017; Kubben et al., 2016), and calcium‐phosphate deposition (Villa‐Bellosta et al., 2013)], of progerin, although agents targeting the DNA repair and damage–response pathways (Aguado et al., 2019; Barcena et al., 2018; Liu et al., 2013), the JAK‐STAT pathway (which are involved in development and homeostasis) (Liu et al., 2019), purine metabolism (which provides basic components for the synthesis of nucleotides) (Mateos et al., 2018), and some conventional age‐associated pathways [e.g., sirtuin pathway (Endisha et al., 2015; Liu et al., 2012; Strandgren et al., 2015), growth hormone (GH)/insulin/IGF‐1 signaling pathway (Barcena et al., 2018; Marino et al., 2010), and AMPK‐TOR pathway (Akinci et al., 2017; Bikkul et al., 2018; Cao et al., 2011; Cenni et al., 2011; Gabriel et al., 2016; Harhouri et al., 2017; Ibrahim et al., 2013; Kawakami et al., 2019; Park & Shin, 2017)] have been adopted. In addition, reactive oxygen species (ROS) generation has been associated with physiological aging, and its inhibition has also been found to be therapeutic in HGPS fibroblasts (Kang et al., 2017; Kubben et al., 2016; Richards et al., 2011). The choice of many of these agents and their corresponding targets may partly be explained by the resemblance of HGPS phenotypes to symptoms of physiological aging.

**TABLE 4:** Biological targets adopted for tackling HGPS

Level	Target	Example	Ref.
Molecular level	Protein prenylation	Pravastatin	Gordon et al. (2016); Wang et al. (2010)
		Zoledronate	Gordon et al. (2016); Wang et al. (2010)
		GGTI‐2147	Mehta et al. (2011)
	Protein farnesylation	FTI‐276	Wang et al. (2010)
		FTI‐277	Bikkul et al. (2018); Mehta et al. (2011); Pacheco et al. (2014)
		Lonafarnib	Gordon et al. (2016); Gordon et al. (2012); Gordon et al. (2018); Ullrich et al. (2013)
		N6‐isopentenyladenosine	Bifulco et al. (2013)
	GH/insulin/IGF‐1 signaling	Recombinant IGF‐1	Marino et al. (2010)
		Methionine‐restrict diet	Barcena et al. (2018)
	Sirtuin pathway	Resveratrol	Liu et al. (2012); Strandgren et al. (2015)
		Plasmids for SIRT6 overexpression	Endisha et al. (2015)
	ROS generation	Y‐27632	Kang et al. (2017)
		NAC	Kubben et al. (2016); Richards et al. (2011)
	Purine metabolism	SAMe	Mateos et al. (2018)
	NF‐κB signaling	siRNA to inhibit ATM expression	Osorio et al. (2012)
		KU55933	Osorio et al. (2012)
		Sodium salicylate	Osorio et al. (2012)
	NRF2 pathway	Oltipraz	Kubben et al. (2016)
		CPDT	Kubben et al. (2016)
		TAT‐14	Kubben et al. (2016)
		AI‐1	Kubben et al. (2016)
		Constitutively activated NRF2	Kubben et al. (2016)
		siRNA to knock down CAND1 expression	Kubben et al. (2016)
		Sulforaphane	Gabriel et al. (2015); Gabriel et al. (2017)
	Calcium‐phosphate deposition	Sodium pyrophosphate tetrabasic decahydrate	Villa‐Bellosta et al. (2013)
	JAK‐STAT pathway	Baricitinib	Liu et al. (2019)
	DNA repair and damage–response pathways	Sequence‐specific telomeric antisense oligonucleotides	Aguado et al. (2019)
		Methionine‐restrict diet	Barcena et al. (2018)
		siRNA targeting Suv39h1	Liu et al. (2013)
	Production and binding of progerin/lamin A	All‐trans retinoic acid	Pellegrini et al. (2015)
		Metformin	Egesipe et al. (2016)
		Therapeutic RNA targeting *LMNA* gene	Beyret et al. (2019); Santiago‐Fernandez et al. (2019)
		Antisense oligonucleotides that reduce prelamin A production	Lee, Nobumori, et al. (2016)
		1α,25‐dihydroxyvitamin D3	Kreienkamp et al. (2016)
		JH1	Lee, Jung, et al. (2016)
		JH4	Lee, Jung, et al. (2016)
		JH13	Lee, Jung, et al. (2016)
		Antisense oligonucleotides that prevent pathogenic *Lmna* splicing	Harhouri et al. (2016); Osorio et al. (2011)
		A helper‐dependent adenoviral vector designed to correct the HGPS mutation	Liu et al. (2011)
	AMPK‐TOR signaling	Rapamycin	Akinci et al. (2017); Bikkul et al. (2018); Cao et al. (2011); Cenni et al. (2011); Kawakami et al. (2019)
		Temsirolimus	Gabriel et al. (2016)
		MG132	Harhouri et al. (2017)
		Lentiviral short hairpin RNA targeting isoprenylcysteine carboxyl methyltransferase	Ibrahim et al. (2013)
		Metformin	Park and Shin (2017)
Cellular level	Nuclear protein export	Leptomycin B	Garcia‐Aguirre et al. (2019)
	Cell senescence	shRNA targeting the phospholipase A2 receptor	Griveau et al. (2018)
		ABT‐737	Ovadya et al. (2018)
		Spermidine	Ao et al. (2019)
		Vitamin C	Geng et al. (2019)
		Quercetin	Geng et al. (2019)
	Autophagy	MG132	Harhouri et al. (2017)
	Telomere functioning	hTERT mRNA	Li et al. (2019)
	Mitochondrial functioning	Methylene blue	Xiong et al. (2016)
		CP‐466722	Kuk et al. (2019)
	Endoplasmic reticulum stress and unfolded protein response	Tauroursodeoxycholic acid	Hamczyk et al. (2019)
	Cellular physiology	Cellular reprogramming mediated by overexpression of Yamanaka factors	Ocampo et al. (2016)
Physiological level	Gut microbiome	Fecal microbiota from healthy subjects	Barcena et al. (2019)
	Bone marrow microenvironment	BRL37344	Ho et al. (2019)
	Vascular calcification	ARL67156	Villa‐Bellosta (2019)
		ATP	Villa‐Bellosta (2019)
		Levamisole	Villa‐Bellosta (2019)

Among different pathways, one of the pathways that are worth highlighting is the AMPK‐TOR pathway, which has been reported by one of the analyzed studies to be possibly activated upon the use of lentiviral short hairpin RNA targeting isoprenylcysteine carboxyl methyltransferase (Ibrahim et al., 2013), leading to the abolishment of the premature senescence of *Zmpste24*‐deficient fibroblasts. Interestingly, the AMPK‐TOR pathway is involved in autophagy. As shown by using MG132 which is therapeutic to HGPS via autophagy enhancement (Harhouri et al., 2017), inhibition of the AMPK‐TOR pathway may serve as a potential path for treatment development. The therapeutic potential of targeting autophagy has been demonstrated by the use of rapamycin, which is a macrolide produced by *Streptomyces hygroscopicus* (Apelo & Lamming, 2016). This agent and its analog, temsirolimus, have been found to enhance progerin clearance through an autophagic mechanism (Akinci et al., 2017; Bikkul et al., 2018; Cao et al., 2011; Cenni et al., 2011; Gabriel et al., 2016; Kawakami et al., 2019) and hence are thought to be drug candidates for the treatment of HGPS. It is, however, worth noting that rapamycin may inhibit adipogenesis (Cho, Park, Lee, Lee, & Kim, 2004), caution should be exercised when rapamycin and its analog are applied because HGPS patients often suffer from lipoatrophy and lipodystrophy. In addition, rapamycin and its analog are inhibitors of the AMPK‐TOR pathway. This pathway is involved in the regulation of multiple biological processes, ranging from protein synthesis and cell proliferation to molecular trafficking and glucose homeostasis. Detailed investigations are needed to determine possible side effects caused by the inhibition of these processes in HGPS patients.

In fact, it may be tempted to believe that confronting progerin at its source is a must for the treatment of HGPS; however, as shown by some of the analyzed studies, tackling the downstream effects of progerin may also result in therapeutic benefits. This has been revealed by the amelioration of progeroid phenotypes upon administration of interventions targeting nuclear protein export (Garcia‐Aguirre et al., 2019), senescence (Ao et al., 2019; Geng et al., 2019; Griveau et al., 2018; Ovadya et al., 2018), telomere elongation (Li et al., 2019), mitochondrial functioning (Kuk et al., 2019; Xiong et al., 2016), endoplasmic reticulum stress (Hamczyk et al., 2019), unfolded protein response (Hamczyk et al., 2019), autophagy (Harhouri et al., 2017), and even the physiology of affected cells (Ocampo et al., 2016), despite the failure of these interventions to correct the disease‐causing mutation. Similar observations have been made at the physiological level. For instance, HGPS is associated with accelerated cardiovascular diseases (e.g., vascular stenosis and excessive vascular calcification). By tackling vascular calcification using combined treatment with ATP, levamisole, and ARL67156 to increase the extracellular pyrophosphate availability, longevity prolongation in the progeroid mouse model has been achieved (Villa‐Bellosta, 2019). Treatment of *Lmna^G609G/G609G^* mice with a β3‐AR agonist, namely BRL37344, has also successfully decreased premature expansion of myeloid cells and hematopoietic stem cells (HSCs) (Ho et al., 2019), suggesting the possibility of ameliorating some of the HGPS symptoms simply by targeting the bone marrow microenvironment. Recently, upon fecal microbiota transplantation from wild‐type mice, the healthspan and lifespan of both *Lmna^G609G/G609G^* and *Zmpste24^−/−^* mice have been shown to be improved, even though the disease‐causing gene mutation has not been corrected (Barcena et al., 2019). All these studies demonstrate that correction of the primary genetic defect underlying the disease is not a prerequisite to attain therapeutic benefits.

## 8 IMPLICATIONS FOR FUTURE RESEARCH

As presented in preceding sections, significant advances in the design of treatment strategies have been made over the last 10 years. Few barriers, however, have still to be addressed in future research before practicable interventions can be achieved. One of the barriers is the lack of strategies for effective systemic delivery. This is particularly important when strategies targeting HGPS at the molecular level are designed, and is also one of the prerequisites for effective implementation of nucleic acid therapy in HGPS patients. Over the last ten years, different therapeutic agents have been exploited for HGPS treatment. Even though further optimization and investigation are needed before these agents can be possibly translated into routine clinical practice, the possibility of eliciting therapeutic effects by these agents implies that the corresponding biological targets are feasible sites of intervention. In another word, other agents that can act on those targets may, at least theoretically, become potential candidates for HGPS treatment.

Another area that is worth paying attention to in the forthcoming decades is the development of more effective and accurate strategies to evaluate the efficiency of a reported HGPS therapy. To accomplish this, not only should we develop a model that enables more accurate prediction of the clinical outcome of a reported intervention, but we should also enhance the comprehensiveness of treatment evaluation. Till now most of the studies in the literature on the treatment of HGPS have only worked on the classic HGPS genotype. The efficiency of reported therapies on individuals with nonclassic HGPS genotypes has rarely been determined. In fact, so far more than 10 different genetic conditions with nucleotide variants in *LMNA* have been documented (Gordon et al., 1993). The phenotypic features of individuals with these nonclassic genotypes as compared to classic HGPS may vary greatly (Gordon et al., 1993). Moreover, variants in *ZMPSTE24* are able to cause HGPS phenotypes (Gordon et al., 1993). The effect of the treatment to individuals with different HGPS genotypes should, therefore, be properly evaluated as this may affect treatment design and implementation.

## 9 CONCLUDING REMARKS

Progress in research on HGPS has led to a rapidly increasing number of therapeutic candidates; however, similar to the case of physiological aging, currently there is no cure for HPGS. In this article, we have systematically retrieved and analyzed 56 articles to examine the latest advances in the development of HGPS treatment over the last ten years. Different biological targets have been presented, along with an evaluation of the opportunities and limitations of diverse existing treatment strategies. Because this article only reviews the advances made over the last ten years, treatment strategies reported before the review period [e.g., correction of the aberrant splicing event using oligonucleotides (Scaffidi & Misteli, 2005), or suppression of proliferative defects by p53 inactivation (Kudlow et al., 2008)], or works contributing to the understanding of the disease [including the development of mouse models showing reversible HGPS phenotypes (Sagelius et al., 2008) and the establishment of the human iPSC model of HGPS (Zhang et al., 2011)] have not been covered by this article. Related advances, however, have been reviewed in the literature (Ashapkin, Kutueva, Kurchashova, & Kireev, 2019; Brassard, Fekete, Garnier, & Hoesli, 2016; Del Campo, Hamczyk, Andres, Martinez‐Gonzalez, & Rodriguez, 2018; Gonzalo & Kreienkamp, 2015; Prokocimer et al., 2013; Trigueros‐Motos, Gonzalez, Rivera, & Andres, 2011). Readers may refer to related articles for details. In summary, HGPS is a progeroid syndrome which has attracted extensive research interest partly because it might provide a window into the mechanism and treatment of physiological aging. Although copious barriers have to be overcome before a cure for HGPS can be developed, with increasing understanding of the molecular mechanism of the disease, more therapeutic targets are expected to be identified. Along with the continuous enhancement in the design of treatment strategies, the emergence of a cure is only a matter of time.

## CONFLICT OF INTEREST

The authors declare that there are no conflicts of interest.
